# Rhizorhabdus antheiae sp. nov. and Sphingomonas eleionomae sp. nov., new aerobic anoxygenic phototrophs isolated from a Manitoban marsh

**DOI:** 10.1099/ijsem.0.007018

**Published:** 2026-01-09

**Authors:** Katia Messner, John A. Kyndt, Vladimir Yurkov

**Affiliations:** 1 Department of Microbiology, University of Manitoba, Winnipeg, Manitoba, Canada; 2 College of Science and Technology, Bellevue University, Bellevue, NE, USA

**Keywords:** aerobic anoxygenic phototrophs, bacteriochlorophyll a, marsh, *Rhizorhabdus*, *Sphingomonas*, *Sphingomonadaceae*

## Abstract

A peach-pigmented strain FW153^T^ and a yellow-pigmented FW199^T^ were isolated from marsh water located at Fort Whyte, Manitoba, Canada. Both produce an anoxygenic photosynthetic apparatus, with a reaction centre encircled by a light-harvesting I complex containing bacteriochlorophyll *a*. They do not produce RuBisCo and do not grow anaerobically or autotrophically, supporting the classification of both as aerobic anoxygenic phototrophs. Sequencing of the 16S rRNA gene showed similarity to *Rhizorhabdus phycosphaerae* (99.93%), *Rhizorhabdus wittichii* (98.73%) and *Rhizorhabdus histidinilytica* (98.52%) for FW153^T^, while for FW199^T^, it was *Sphingomonas molluscorum* (97.47%), *Sphingomonas dokdonensis* (97.44%) and *Sphingomonas kyeonggiensis* (97.08%). Polyphasic analysis identified differences in physiology and cellular fatty acid composition, as well as within the genome, with average nucleotide identity (<95%) and digital DNA–DNA hybridization values (<70%) between each strain and their closest relatives supporting species designation. Therefore, we propose that FW153^T^ (=NCIMB 15610^T^=DSM 120042^T^) and FW199^T^ (=NCIMB 15611^T^=DSM 120043^T^) be classified as the type strains of new species with the names *Rhizorhabdus antheiae* sp. nov. and *Sphingomonas eleionomae* sp. nov., respectively.

## Introduction

Wetlands are an imperative component of the landscape in Manitoba, Canada, comprising ~43% of the total land area [1]. They serve as a biodiversity hotspot, being the exclusive home of many waterfowl, fish and plants [2,3]. Furthermore, they act as a temporary reservoir during flooding [2], protecting surrounding communities, and as a carbon sink countering greenhouse gas emissions [2], which highlights their impact locally and globally. Marshes are a type of wetland that does not form peat, usually do not have trees or shrubs and tend to be found near shallow open waters [2]. In Winnipeg, Manitoba, there is a marsh located on FortWhyte Alive, a protected habitat that is a recreational and educational site. Previously, sampling was conducted here, as well as in other marshes, to assess the diversity of the anoxygenic phototrophic communities [4]. As a result, many bacterial strains were isolated, including FW153^T^ and FW199^T^, which were identified as aerobic anoxygenic phototrophs (AAP) [4].

AAP are mixotrophic bacteria, capable of aerobic cellular respiration, as well as photosynthesis to fuel the cell. Unlike traditional anaerobic anoxygenic phototrophs, AAP use it aerobically as a supplement to their primarily chemoheterotrophic metabolism [5]. They are unable to grow autotrophically and do not contain RuBisCo [6]. Energy acquired from light helps them grow competitively alongside other heterotrophs, as well as survive starvation conditions [7].

*Sphingomonadaceae* is a family in *Alphaproteobacteria* which comprises members that are aerobic or facultative anaerobes; divide by binary fission, budding or asymmetrically; contain ubiquinone-10 as its primary respiratory quinone; and are diverse in colour ranging from orange-brown to various shades of yellow to non-pigmented [8]. After an emendation to the group [9], the family, as of writing, contains 19 validly published genera [10]. Recently, additional changes have been proposed to the family alongside the entire order (*Sphingomonadales*), which proposes additional new taxa [11]. Of the validly published genera in *Sphingomonadaceae*, *Rhizorhabdus* and *Sphingomonas* are closely related. *Sphingomonas* is an incredibly large genus, currently containing 181 validly published species, while *Rhizorhabdus* is comparatively very small, with only six members [10], three of which were initially classified as *Sphingomonas* [9]. Here, we describe a new feature in common with the two genera, which is comprising members that are AAP. While this has been identified in *Sphingomonas* spp. previously [12,14], the production of anoxygenic photosynthesis apparatus had yet to be seen in *Rhizorhabdus*. Here, we report FW153^T^ and FW199^T^, which represent two new species for which the names *Rhizorhabdus antheiae* and *Sphingomonas eleionomae*, respectively, are proposed.

## Methods

### Isolation and cultivation

Strains FW153^T^ and FW199^T^ were isolated on potato media (PM) and rich organic (RO) media, respectively, from marsh water collected at FortWhyte Alive, Winnipeg, Manitoba, Canada [4]. PM contains the following (g/L) [5]: Na-acetate, 0.5; yeast extract, 1; potato broth (500 ml); trace elements (TE) solution [15], 2 ml; and vitamin solution (VS) [15], 2 ml. The RO media comprises the following (g/L) [16]: MgCl_2_, 0.5; K_2_PO_4_, 0.3; NH_4_Cl, 0.3; KCl, 0.3; CaCl_2_, 0.05; Na-acetate, 1; yeast extract, 1; bactopeptone, 0.5; casamino acids, 0.5; TE, 2 ml; and VS, 2 ml. Each was grown on RO for 7 days in the dark at 28 °C on a shaking incubator for subsequent characterization, unless otherwise indicated. For long-term storage, cells were cryopreserved at −80 °C in 30% (v/v) glycerol and modified RO with 10% organics (g/L): yeast extract, 0.1; Na-acetate, 0.1; bactopeptone, 0.05; and casamino acids, 0.05.

### Phenotypic properties

Cell shape and size were determined after 2 days of growth. Motility was checked after 1, 2, 3 and 6 days of growth via phase contrast microscopy (Zeiss Axioskop 2, Jena, Germany). Gram staining [17] was performed alongside a KOH hydrolysis [18] for confirmation. Colony morphology was evaluated after 7 days.

Temperature and pH range; oxidase, catalase, nitrate reduction and indole tests; hydrolysis of Tweens 20, 40, 60 and 80, starch, gelatin and agar; antibiotic susceptibility; photo- and chemo-heterotrophic anaerobic growth; fermentation of fructose, glucose and sucrose; and aerobic photoautotrophy were previously assessed [4]. Tolerance of NaCl was determined by growing cells in 0% to 5.0% with 0.5% increments. The utilization of complex (bactopeptone, casamino acids and yeast extract) and single carbon sources such as organic Na salts (acetate, butyrate, citrate, formate, glutamate, lactate, malate, pyruvate and succinate), sugars (fructose, glucose, lactose and sucrose), amino acid (glycine), sugar alcohols (glycerol and sorbitol) and simple alcohols (ethanol and methanol) was tested at 0.5% concentration in liquid RO modified to exclude any other organics. API 20 NE and API ZYM strip tests (bioMérieux, France) were run following the manufacturer’s instructions to assess the usage of other carbon sources and production of additional enzymes.

To assess the photosynthetic complex expression conditions for strains FW199^T^ and FW153^T^, they were grown for 5 days on RO plates in constant light or in the dark. Whole cells and pigment extracts were prepared for absorption spectra reading in the 300–1,100 nm region as described [19].

FW153^T^ was grown on R2A plates for 2 days at 30 °C in the dark, while FW199^T^ was grown on tryptic soy agar plates at 28 °C for 2 days. Triplicates of cells were collected, lipid extractions were conducted and purification was performed using Folch’s method [20]. Samples were analysed with GC to identify fatty acid composition.

### Sequencing and phylogeny

The 16S rRNA gene fragments covering the V1-V8 region were obtained from extracted DNA via Sanger sequencing with universal primers 27F (5′-AGAGTTTGATCCTGGCTCAG-3′) and 1492R (5′-GGTTACCTTGTTACGACTT-3′). Resulting chromatograms were processed on DNA Baser Assembler v4.36.0 (Heracle BioSoft SRL, Mioveni, Romania). Type species with the most similar sequence were identified through a standard nucleotide blast search [21]. A 16S rRNA-based phylogenetic tree was constructed with mega 12 software [22], using the maximum-likelihood method and 1,000 bootstrap replicates. The Kimura 2-parameter model [23] selected for the tree was determined with the mega X ‘find best DNA model’ tool. To measure evolutionary rate differences among sites, a gamma distribution (+G, parameter=0.6495) with five rate categories was applied, and it was assumed that a certain fraction of sites are evolutionarily invariable ([+I], 76.40% sites). The final dataset had 44 nucleotide sequences and 1,532 positions.

To obtain the whole-genome sequence, DNA was extracted and sequenced with the Illumina MiniSeq system as reported [19]. For strain FW153^T^, this generated 3,567,588 reads comprising 282.41 Mbp, and for strain FW199^T^, 2,507,296 reads comprising 201.21 Mbp were obtained. The dataset was quality-checked with FASTQC (version 1.0.0) and cleaned with FASTQ Toolkit (version 2.2.6), assembled *de novo* using Unicycler (version 0.5.0) [24] and annotated with the Prokaryotic Genome Annotation Pipeline [25] as described [19]. G+C content (mol%) was determined from the genome sequence. Genome quality was assessed using CheckM (version 1.2.3) [26]. For phylogenomic tree inference, nucleotide sequences of 92 conserved marker genes were extracted from the genomes of 35 type strains of species in *Sphingomonas* and *Rhizorhabdus* using the UBCG v. 3 pipeline [27]. These aligned marker gene sequences were subjected to concatenation and partitioning with FASconCAT-G (version 1.06) [28]. A maximum-likelihood phylogeny was then inferred from the concatenated data matrix with edge-unlinked partitions, and branch support was inferred from 1,000 replicates with non-parametric bootstrapping on IQ-TREE 3 (version 3.0.1) [29] with the model determined using ModelFinder [30]. The tree was visualized with iTOL [31]. Digital DNA–DNA hybridization (dDDH) and CIs were calculated with formula d_4_ of the Genome-to-Genome Distance Calculator (version 4.0) applying the recommended settings on the Type (Strain) Genome Server [32,33]. Average nucleotide identity (ANI) was assessed with JSpeciesWS [34].

## Results and discussion

### Spectral analysis

**Fig. 1.**
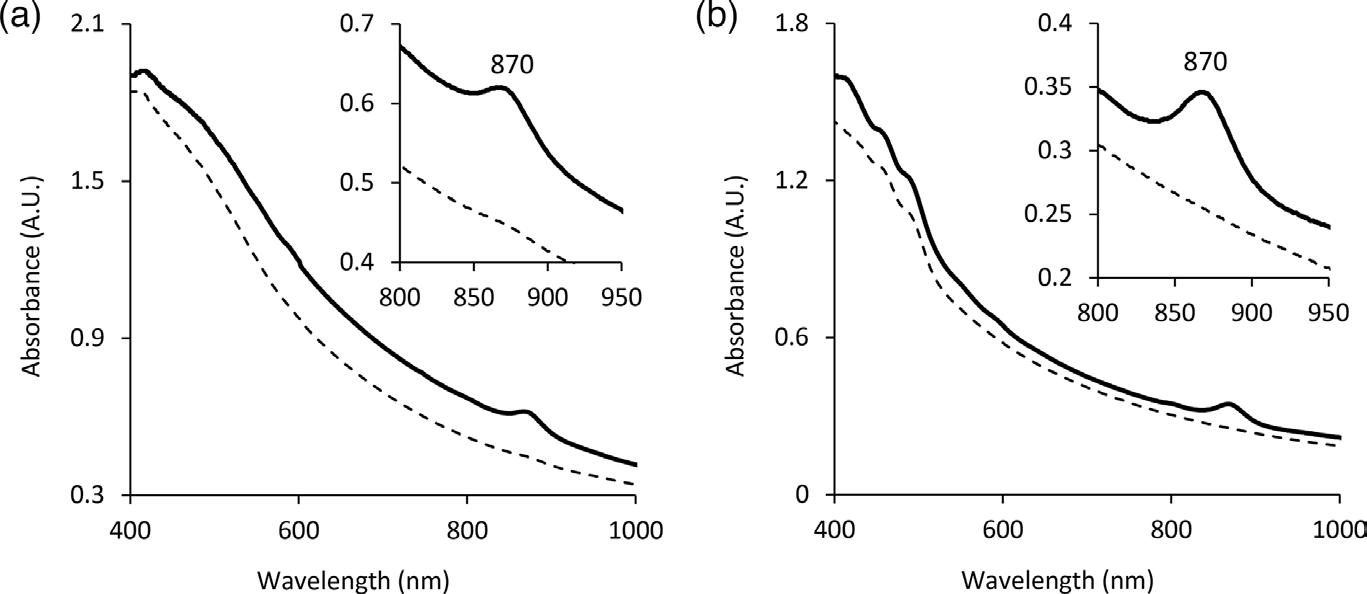
Absorbance spectra of whole cells of FW153^T^ (**a**) and FW199^T^ (**b**) grown in the dark (bold line) and in the light (dashed line). Wavelengths where significant peaks are observed are indicated.

The whole-cell absorption spectra of strains FW199^T^ and FW153^T^ have a single peak at 870 nm (Fig. 1). This corresponds to absorbance by a light-harvesting I complex, indicating that they likely have an aerobic anoxygenic photosynthetic apparatus. The absorbance peak is quite small compared to what can be seen in other AAP; however, this may be a result of the expression being tightly regulated and photosynthesis being used exclusively for starvation conditions [7]. The primary pigment needed for this process, bacteriochlorophyll *a* (Bchl *a*), is produced in both strains, as can be detected in the pigment extracts at 770 nm (Fig. S1, available in the online Supplementary Material). In these absorbance spectra, carotenoid production can also be seen as a series of peaks in the 400–550 nm region, which gives each strain their respective colour (Fig. S1) [5]. Both strains were grown in the dark, as well as in light to evaluate the regulation of photosynthetic complex production. Like the majority of AAP, they only synthesize the photosynthetic apparatus in the dark, as indicated by the lack of Bchl *a* peaks in both whole cell and pigment extract spectra of light-grown cells (Figs 1 and S1) [5,35]. While there have been other *Sphingomonas* spp. that have been classified as AAP [12,14], making FW199^T^ a new member of that group, FW153^T^ is the first identified AAP among *Rhizorhabdus*. *Rhizorhabdus phycosphaerae* also has the same photosynthesis genes as FW153^T^, but previous analysis did not find any Bchl *a* produced [36].

### Morphology, physiology and chemotaxonomy

**Fig. 2.**
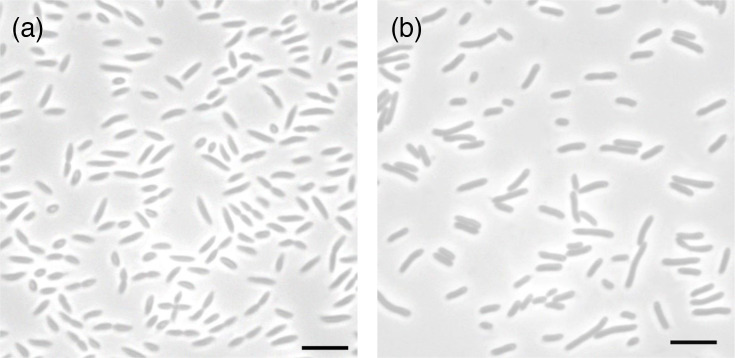
Cells of FW153^T^ (**a**) and FW199^T^ (**b**). Bars are 5 µm.

FW153^T^ forms peach-coloured, circular (2 mm), convex, translucent and glossy colonies that have a soft texture when grown on RO plates for a week. It forms tapered rods that are 2.9–3.3 µm in length and 0.6–0.7 µm in width (Fig. 2). They match the cell morphology in *R. phycosphaerae* but are much longer in length (Table 1). Cells are Gram-negative and only motile in early stages of growth (after a day). FW153^T^ is a mesophile, an alkalitolerant neutrophile and halotolerant. Its optimum growth temperature is 32 °C; however, it has a wider growth range at 4–41 °C, similar to *Rhizorhabdus histidinilytica* (Table 1). The optimal pH is 6; however, it can grow up to pH 9. Although FW153^T^ was isolated from a freshwater marsh, it tolerates NaCl up to 2.5%. It is an obligate aerobic heterotroph, unable to grow in the anaerobic and photoautotrophic conditions tested. With this being the case, FW153^T^ is an AAP as it requires organics to grow, cannot survive in non-oxygenated environments and uses an anoxygenic photosynthetic apparatus. Based on individual testing and the API 20 NE strips, it metabolizes all tested complex and the following single carbon sources: acetate, butyrate, glucose, glutamate, pyruvate and sucrose. It was unable to use the remaining carbons included in the API 20 NE and all sugar alcohols and simple alcohols tested, as well as citrate, formate, fructose, glycine, lactose, lactate, malate and succinate. The API ZYM revealed that cells produce all enzymes listed except lipase (C14), cystine arylamidase, α-galactosidase, β-glucuronidase, α-mannosidase and α-fucosidase. The cells are also positive for oxidase, catalase and nitrate reductase, but negative for indole, amylase, gelatinase, urease and arginine dihydrolase. They were unable to hydrolyse Tweens 20, 40, 60 and 80. FW153^T^ was susceptible to chloramphenicol, imipenem, kanamycin, polymyxin B and tetracycline but resistant to ampicillin, erythromycin, penicillin G, nalidixic acid and streptomycin. The major lipids in FW153^T^ are C_18 : 1_ ω7c, C_16 : 1_ and C_16 : 0_. While C_18 : 1_ ω7c and C_16 : 0_ are described as the major fatty acids in other *Rhizorhabdus*, C_16 : 1_ has not been seen to comprise a major component in the other species [36,39], forming a key distinguishing feature.

**Table 1.** Differential characteristics of strain FW153^T^ from closely related *Rhizorhabdus* and type species of genus [36,37, 47, 48]

**Species**	*R. antheiae*	*R. phycosphaerae*	*R. wittichii*	*R. histidinilytica*	*R. argentea*
**Strain**	**FW153^T^**	**MK52^T^**	**RW1^T^**	**UM2^T^**	**SP1^T^**
Isolation environment	Marsh water, Winnipeg, Canada	Phycosphere of *Microcystis aeruginosa*	Water of the River Elbe	Hexachlorocyclohexane dump site, India	Soil, Montes de Toledo, Spain
Colony colour	Peach	Creamy pink	Yellow	Cream	White
Genomic evidence of photosynthesis	+	+	−	−	−
Bchl *a*	+	−	−	−	−
Cell shape	Tapered rod	Tapered rod	Rod	Rod	Rod
Cell size (µm)	2.9–3.3×0.6–0.7	0.5–0.8×1.1–1.4	0.4–0.6×0.9–1.5	1.4×0.4	1.5×0.8
Motility	−	−	+	+	−
Temperature range (°C)	4–41	21–34	27–33	4–40	30–37, min nd
pH range	6–9	5–8	5–8	7.5	6–8
NaCl range (%)	0–2.5	0–2	0–0.1	1–2	nd
**Enzyme activity:**					
Oxidase	+	+	+	−	+
Nitrate reductase	+	−	−	+	−
Aesculin	+	+	+	−	−
Gelatinase	−	−	+	−	nd
Amylase	−	+	−	−	nd
**Utilization of:**					
Arabinose	−	−	−	+	−
Glucose	+	+	+	−	−
Mannose	−	−	+	+	+

All strains are Gram-negative, strict aerobes and positive for catalase.

+, positive or growth observed; −, negative or no growth; nd, not determined.

When grown on solid RO for a week, FW199^T^ forms yellow, circular (1–2 mm), flat, translucent and shiny colonies with a soft texture. Cells are Gram-negative, motile (after 1 and 2 days of growth) and rod-shaped, specifically 2.8–4.4 in length and 0.6–0.7 in width (Fig. 2). Overall, the cell and colony morphology match what is observed in *Sphingomonas* (Table 2). FW199^T^ grows optimally at 32 °C, pH 6 and 0% NaCl. The range is 20–41 °C, making it less tolerant to the colder and more to warmer temperatures compared to the closest relatives (Table 2). It can survive up to pH 10 and a 4.5% NaCl concentration. This halotolerance is considerably higher than in FW153^T^, which was isolated from the same site; however, it is comparable to *Sphingomonas molluscorum* and *Sphingomonas dokdonensis* (Table 2). Like FW153^T^, it could neither grow autotrophically nor anaerobically, supporting its designation as an AAP. FW199^T^ can use arabinose, all tested complex sources, fructose, glucose, glutamate, lactose, maltose, mannose, *N*-acetylglucosamine and sucrose as a single carbon source. It was unable to use any of the alcohols or the other organic salts assessed, glycine and the remaining carbons on the API 20 NE. Individual enzyme testing and API ZYM showed that catalase, oxidase, amylase, valine arylamidase, trypsin, acid phosphatase, alkaline phosphatase, esterase lipase (C8), leucine arylamidase, *α*-chymotrypsin, β-galactosidase, α-glucosidase, β-glucosidase, *N*-acetyl-β-glucosaminidase and naphthol-AS-BI-phosphohydrolase are produced by FW199^T^. The cells hydrolyse Tweens 20, 60 and 80, but not 40. The strain was susceptible to erythromycin, imipenem, kanamycin, polymyxin B and tetracycline but not ampicillin, chloramphenicol, penicillin G, nalidixic acid and streptomycin. The major lipids for FW199^T^ are C_18 : 1_ ω7c, C_16 : 1_ and C_16 : 0_ [29,32]. The full fatty acid composition of FW199^T^ and FW153^T^ is in Table S1.

**Table 2.** Differential traits of strain FW199^T^ from closely related *Sphingomonas* and type species of genus [49,53]

**Species**	*S. eleionomae*	*S. molluscorum*	*S. dokdonensis*	*S. kyeonggiensis*	*S. paucimobilis*
**Strain**	**FW199^T^**	**An 18^T^**	**DS-4^T^**	**THG-DT81^T^**	**CL1/70^T^**
Isolation environment	Marsh water, Canada	Marine bivalve (*Anadara broughtoni*), Russia	Soil, Republic of Korea	Soil, Republic of Korea	Respirator, England
Genomic evidence of photosynthesis	+	−	−	−	−
Bchl *a*	+	−	−	−	−
Cell size (µm)	2.8–4.4×0.6–0.7	0.4–0.6×1.0–1.5	0.4–0.6×0.8–3.5	0.5–0.7×1.2–1.5	1.4×0.7
Motility	+	−	+	+	+
Temperature range (°C)	20–41	7–37	10–34	15–30	25–37
NaCl range (%)	0–4.5	0–4	0–5	0–0.5	0
**Enzyme activity:**					
Amylase	+	−	+	−	−
Tween 80	+	+	+	−	+
**Utilization of:**					
Arabinose	+	+	−	+	+
Gluconate	−	+	−	−	−
Glucose	+	+	−	+	+
Maltose	+	+	−	+	+
Malate	−	−	−	+	+
Sucrose	+	−	−	+	+

All strains are Gram-negative, form yellow colonies, are rod-shaped, strict aerobes and are catalase- and oxidase-positive.

+, positive or growth observed; −, negative or no growth.

The results obtained show that both strains FW153^T^ and FW199^T^ are AAP. They produce an anoxygenic photosynthesis complex and are strict aerobes. Autotrophic growth was not observed, confirming that they are obligate heterotrophs. AAP do not fix CO_2_ through the Calvin cycle, due to the absence of RuBisCo [6]; however, they perform anaplerotic reactions to replenish the TCA cycle [6]. Alone, it is not sufficient for survival. Therefore, strains FW153^T^ and FW199^T^, like other AAP, use photosynthesis as an additional source of ATP in conjunction with aerobic respiration [40]. The cyclic anoxygenic photosynthesis pathway generates a proton motive force across the inner membrane via the Q cycle, which then triggers ATP synthesis [40]. While AAP rely on organics for growth, the use of light as an additional source of energy has been observed to help them survive starvation and oligotrophic conditions [7,41]. Photosynthesis allows the cell to direct more of its organic resources for other essential processes rather than ATP generation [42]. Despite it not being a primary fuel resource, the ability for photosynthesis provides both strains an advantage over other typical heterotrophs. Thus, the photoheterotrophic growth makes an important contribution to strain FW153^T^ and FW199^T^’s physiology and survival in the natural environment.

### Phylogeny and genome analysis

**Fig. 3.**
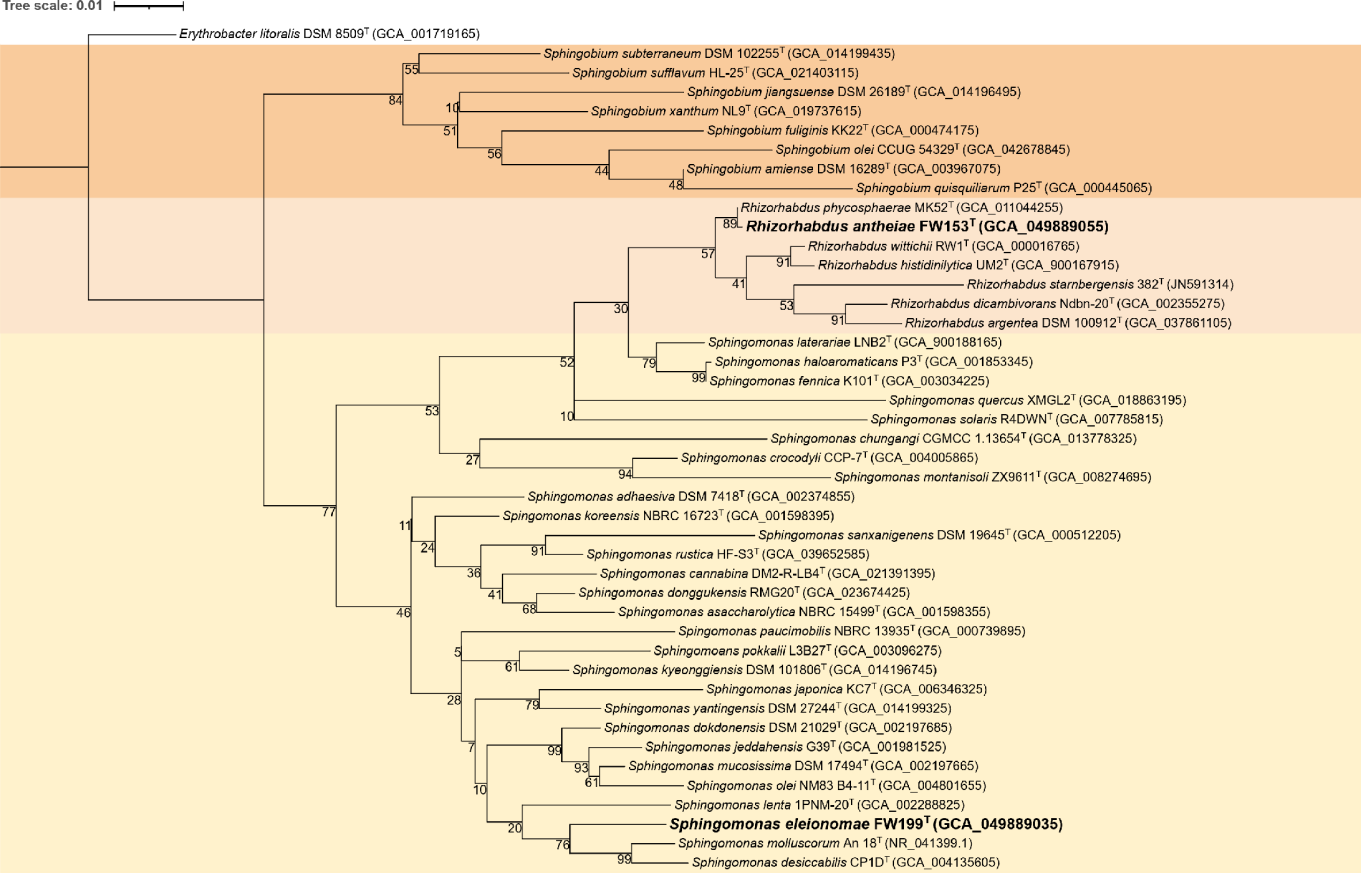
Phylogenetic tree based on 16S rRNA gene of FW153^T^ and FW199^T^ and closely related representatives of the *Sphingomonadaceae*. Different genera are coloured: *Sphingobium* (orange), *Rhizorhabdus* (peach) and *Sphingomonas* (yellow). Version with the highest log likelihood (−7744.46) is shown and drawn to scale. Branch lengths measured in number of substitutions per site. The percentage of trees in which the associated taxa clustered together is shown next to the branches. Accession numbers for isolated 16S rRNA sequences or GenBank Assembly Accession numbers of genome for the 16S rRNA gene are included in parentheses. *Erythrobacter litoralis* DSM 8509^T^ is used as the outgroup.

The near-complete 16S rRNA gene sequence was obtained for both FW153^T^ and FW199^T^ via Sanger sequencing (accession numbers PX123967 and PP726896). Both full sequences of the genes have been located in the genome (1,493 bp for FW153^T^ and 1,494 bp for FW199^T^). According to standard nucleotide blast results of the 16S rRNA gene sequence, the closest relatives to FW153^T^ are *R. phycosphaerae* (99.93%), *R. wittichii* (98.73%) and *R. histidinilytica* (98.52%). The *Sphingomonas* members which share the closest 16S rRNA gene similarity to FW199^T^ are *S. molluscorum* (97.47%), *S. dokdonensis* (97.44%) and *S. kyeonggiensis* (97.08%). The identified close relatives are supported by the general physiological, morphological and chemotaxonomic features. Using these 16S rRNA gene sequences and from other closely related members of *Sphingomonadaceae*, a maximum-likelihood tree was constructed (Fig. 3). Although FW199^T^ shares some features with the neighbouring species, it forms a distinct lineage in *Sphingomonas*. FW153^T^ closely aligns with *R. phycosphaerae* (Fig. 3), despite their phenotypic differences (Table 1). A notable observation that aligns with recent genomic studies [11] is that some *Sphingomonas* spp. more closely align with *Rhizorhabdus*.

**Fig. 4.**
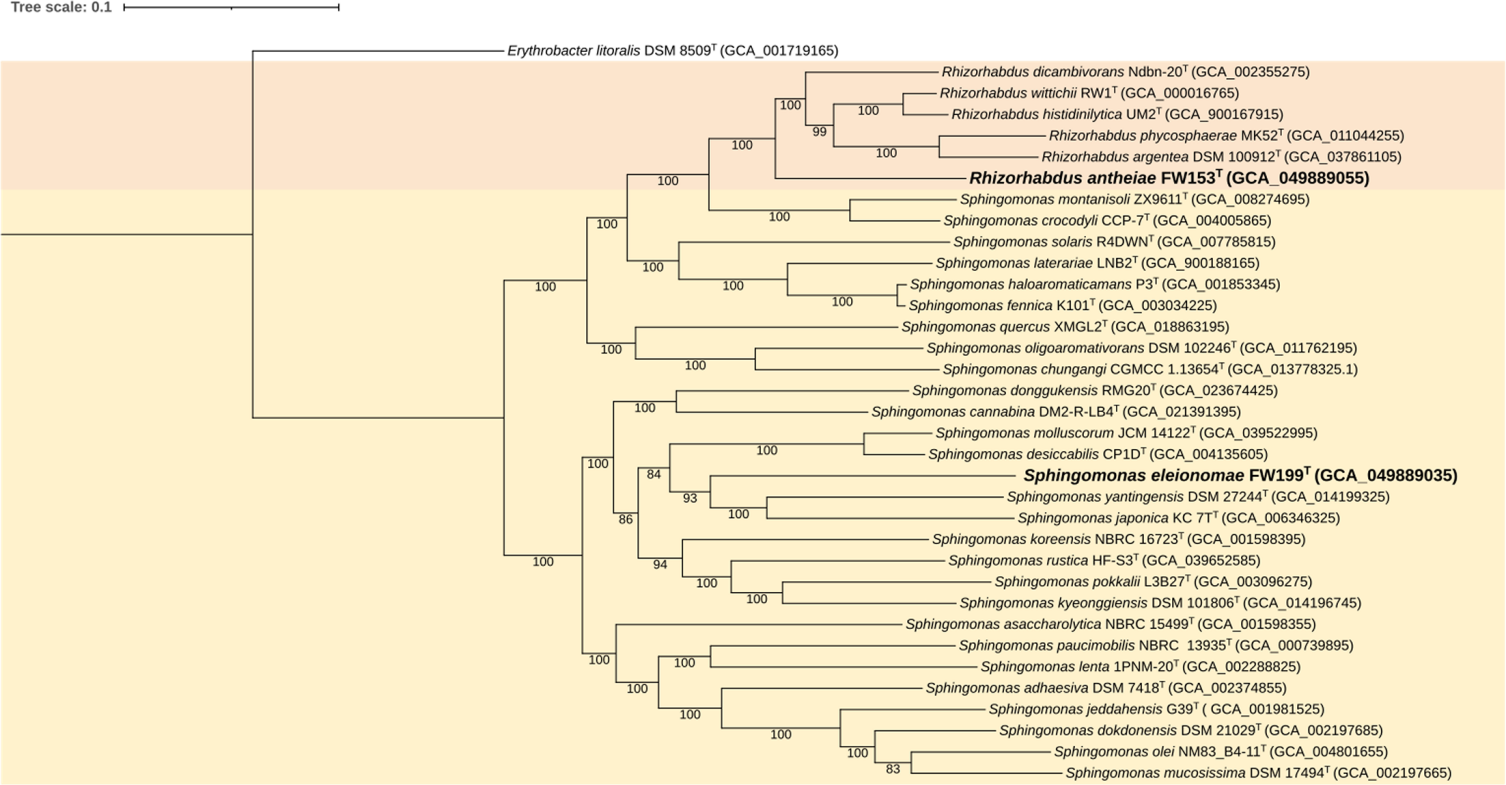
Phylogenomic tree of FW153^T^ and FW199^T^ and selected type strains of species in *Rhizorhabdus* (peach) and *Sphingomonas* (yellow). Version with the highest log likelihood (−1478996.91) is shown and drawn to scale. The branch support values were generated from 1,000 rounds of bootstrapping. Tree scale was defined as the mean number of nucleotide substitutions per site. The GenBank assembly accession number is included in parentheses. *E. litoralis* DSM 8509^T^ is used as the outgroup.

Alongside the 16S rRNA gene phylogenetic tree, a genome-based tree was created using the genomes of *Sphingomonas* and *Rhizorhabdus* species to better resolve relations (Fig. 4). Both formed a clear divergence from other members, further supporting the classification of FW199^T^ and FW153^T^ as new species in *Rhizorhabdus* and *Sphingomonas*.

**Fig. 5.**
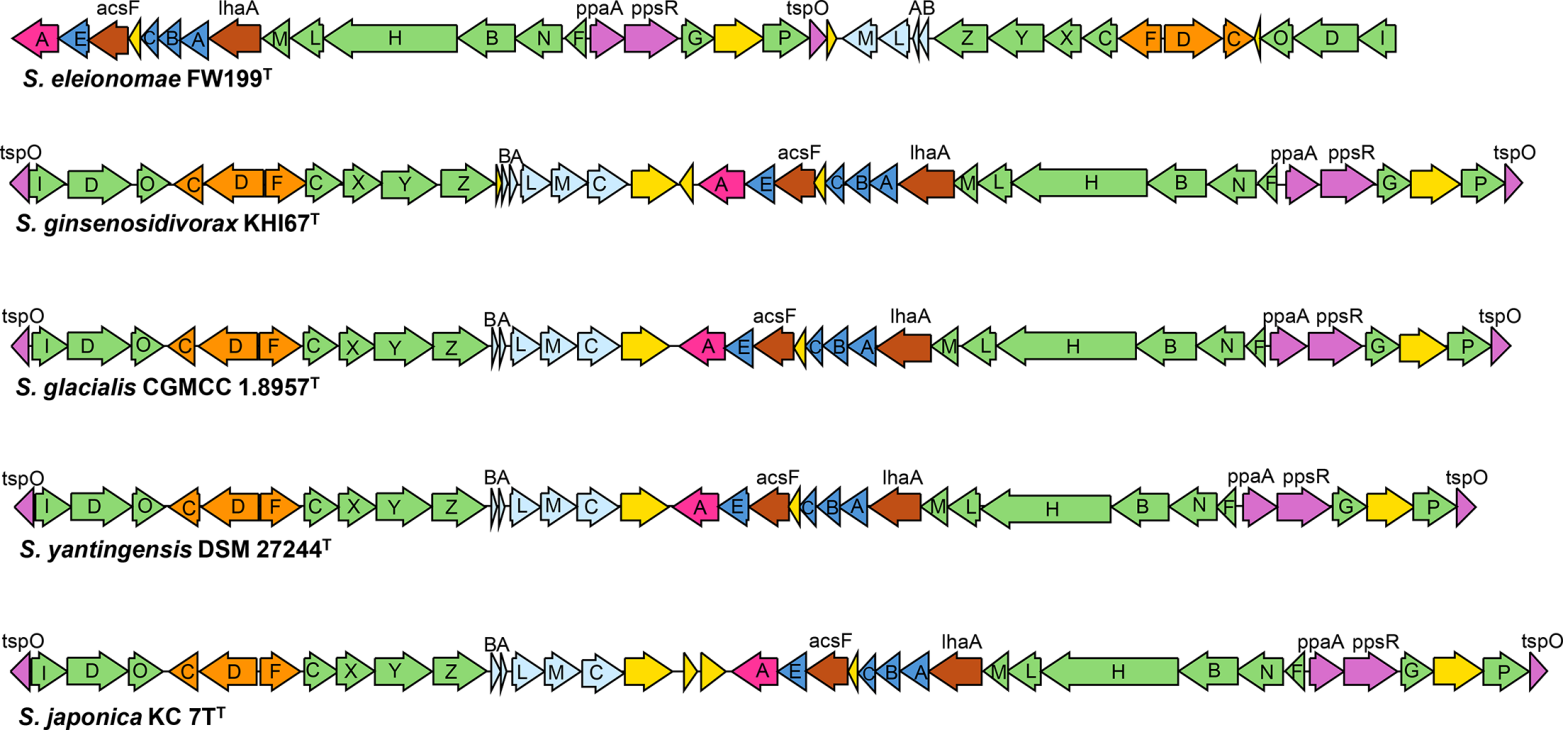
The photosynthetic gene cluster of FW199^T^ and FW153^T^ compared to other AAP in *Sphingomonas* and *Rhizorhabdus*. Genes are coloured as follows: green (*bch*), orange (*crt*), light blue (*puf*), blue (*puh*), pink (*hem*), purple (regulatory genes), brown (other photosynthesis genes) and yellow (hypothetical proteins, uncertain or unrelated genes). GenBank genome accession numbers of strains (starting at top row): GCA_049889035.1, GCA_014653575.1, GCA_006346325.1, GCA_049889055.1 and GCA_011044255.1.

The genome of strain FW153^T^ is 4,286,739 bp, with a 65.54 mol% G+C content and 4,077 annotated protein CDSs (Table 3). It comprises 31 contigs and a 123× average fold coverage. CheckM completeness is 100%, and contamination is 2.45%. No plasmids were identified. The photosynthetic genes are arranged in a cluster (PGC) (Fig. 5), and the genome does not contain RuBisCo, for carbon fixation, further supporting the classification as an AAP. *R. phycosphaerae* also comprised a PGC in the same organization and composition, suggesting that it was previously not grown in the conditions that would induce photosynthesis apparatus expression [28]. The genome was used to infer certain chemotaxonomic features. Strain FW153^T^ can only produce ubiquinone-10 based on the presence of decaprenyl diphosphate synthase in the genome and the absence of other annotated enzymes which encode tail length variants. Genes to produce the following polar lipids were identified: phosphatidylethanolamine, phosphatidyldimethylethanolamine, phosphatidylmethylethanolamine, phosphatidylcholine and phosphatidylglycerol. FW153^T^ had 105 genes which are not found in any of the other sequenced *Rhizorhabdus* spp. Most annotated genes were hypothetical or encoding uncharacterized proteins; however, a couple were noteworthy. FW153^T^ encodes the genes for opine oxidase subunits A, B and C, as well as potential ABC opine transporters. This suggests that, like other bacteria, most notably *Agrobacterium* species [43,44], FW153^T^ is able to use opines as a carbon and nitrogen source. Multidrug efflux pump EmrAB-TolC, a Class A beta-lactamase, as well as a mutation in the S12p protein, was found in the genome, which could possibly explain antibiotic resistance observed in nalidixic acid, penicillin G, ampicillin and streptomycin, respectively.

**Table 3.** Genome features of strain FW153^T^ compared to related members

**Species**	*R. antheiae*	*R. phycosphaerae*	*R. wittichii*	*R. histidinilytica*
Strain*	**FW153^T^**	**MK52^T^**	**RW1^T^**	**UM2^T^**
16S rRNA gene similarity (%)†	100	99.93	98.73	98.52
Genome size (Mb)	4286739	4327794	5915246	5473339
G+C content (mol%)‡	65.54	65.50	67.50	67.35
No. of contigs	31	1	3 (2 plasmids)	59
L50	4	1	1	5
N50	319157	4327794	5382261	409683
No. of CDS	4077	4040	5470	5339
No. of tRNA operons	45	47	48	47
ANI (%)†	100	86.0	78.4	78.6
dDDH (%)†	100	31.5	23.8	23.4

*GenBank genome accession numbers of strains from left to right: GCA_049889055.1, GCA_011044255.1, GCA_000016765.1 and GCA_900167915.1.

†Compared to strain FW153T.

‡Based on genome sequence.

Strain FW199^T^ genome is 3,088,356 bp, with a G+C content of 65.43 mol% and 2,912 annotated protein CDSs (Table 4). It has a 120× average fold coverage of its 12 contigs, and no plasmids were identified. CheckM completeness and contamination are 98.62 and 0.55%, respectively. AAP designation is further evidenced by the identification of the PGC and absence of RuBisCo. FW199^T^ PGC is unlike other phototrophic *Sphingomonas* as it does not contain a cytochrome *c* subunit as part of its reaction centre and has a distinct organization. Relative to other *Sphingomonas*, the *bchIDO-crtCDF-bchCXYZ-pufLMAB* region is inverted and located behind the *bchP-tspO* section (Fig. 5). When identifying the most similar sequences of the *pufLM*, as well as the *ppsR* and *puhA* genes, using blast, the most similar sequences (top 5) were not *Sphingomonas*. Interestingly, they were from other genera in *Sphingomonadaceae* or *Erythrobacteraceae* and had a similar organization to what is seen in FW199^T^ (Fig. S2). When an analogous investigation was done for FW153^T^, the sequences of those same genes closely aligned with the phylogeny assessed via 16S rRNA and genome-based trees. This suggests that FW199^T^’s photosynthetic capabilities were acquired through lateral gene transfer and is likely not a result of an inversion within the genome. As with FW153^T^, the genome of FW199^T^ was investigated to infer some chemotaxonomic traits. Strain FW199^T^ contains decaprenyl diphosphate synthase and no other tail length gene variant; therefore, Q-10 is the main ubiquinone. The following polar lipids may be produced: phosphatidylethanolamine, cardiolipin, phosphatidylglycerol and sphingoglycolipids. When FW199^T^ was compared to the closest related *Sphingomonas* based on 16S rRNA, as well as the three most aligned from the genome-based phylogenetic tree, it was found that it contained 90 unique genes, the majority of which were annotated with an unknown function. Antibiotic resistance genes/mutations in FW153^T^ were identified in FW199^T^.

**Table 4.** Genome features of strain FW199^T^ compared to related *Sphingomonas*

**Species**	*S. eleionomae*	*S. molluscorum*	*S. dokdonensis*	*S. kyeonggiensis*
Strain*	**FW199^T^**	**An 18^T^**	**DS-4^T^**	**THG-DT81^T^**
16S rRNA gene similarity (%)†	100	97.47	97.44	97.08
Genome size (Mb)	3088356	3600510	3452664	5517776
G+C content (mol%)‡	65.43	67.81	66.46	66.76
No. of contigs	12	30	16	8
L50	2	3	2	2
N50	822262	435498	739540	1461588
No. of CDS	2912	3414	3366	5089
No. of tRNA operons	46	48	49	54
ANI (%)†	100	72.7	72.2	72.5
dDDH (%)†	100	19.3	20.4	19.7

*GenBank assembly accession numbers of strains from left to right: GCA_049889035.1, GCA_039522995.1, GCA_002197685.1 and GCA_014196745.1.

†Compared to strain FW199T.

‡Based on genome sequence.

Using the genome of FW153^T^, FW199^T^ and their respective closely related members, the ANI and dDDH were assessed. All ANI values were below the 95% cutoff, and all the dDDH were below the 70% barrier for species differentiation [45,46], supporting their classification as new species (Tables 3 and 4).

## Conclusion

Based on the polyphasic analysis, FW153^T^ and FW199^T^ are both new members of *Sphingomonadaceae*, specifically as new species in *Rhizorhabdus* and *Sphingomonas* genera, respectively. FW153^T^ represents the first confirmed AAP in *Rhizorhabdus*. Despite a high 16S rRNA similarity to *R. phycosphaerae*, there are a couple of key features which unequivocally support the designation as a new species. ANI and dDDH, which fall below values for strains to be classified in the same species and phylogenomic branching, show that FW153^T^ and *R. phycosphaerae* are more distantly related than some other members of *Rhizorhabdus* (Fig. 4). Furthermore, there are phenotypic differences as well: cells of FW153^T^ are larger than *R. phycosphaerae*, enzyme produced varies (nitrate reductase, amylase) and FW153^T^ has been shown to produce photosynthetic complexes and Bchl *a*. Phylogenetic trees, carbon sources utilized and enzyme production also show that FW199^T^ diverges from other species in *Sphingomonas*. Therefore, we propose the new names *R. antheiae* and *S. eleionomae* with FW153^T^ and FW199^T^ as the type strains.

## Description of *Rhizorhabdus antheiae* sp. nov.

*Rhizorhabdus antheiae* (an.thei'ae. Gr. fem. n. *Antheia*, the Greek goddess of swamps and marshes; N.L. gen. n. *antheiae*, of Antheia, referring to marsh habitat).

Cells are Gram-negative, motile rods with tapered ends that are ~2.9–3.3 µm in length and 0.6–0.7 µm in width. The strain froms colonies that are peach, circular (2 mm), convex, translucent and glossy which have a soft texture when grown on RO plates for a week. Cells are obligately aerobic and heterotrophic. Growth occurs in the following conditions (optimum): between 4 and 41 °C (32 °C), pH of 6–9 (6) and NaCl (% w/v) up to 2.5% (0%). Bchl *a*, carotenoids, photosynthetic reaction centre and light-harvesting I complex are produced, indicative of aerobic anoxygenic photosynthesis. RuBisCo was not detected in the genome. It produces oxidase, catalase, nitrate reductase, trypsin, acid phosphatase, alkaline phosphatase, cystine arylamidase, esterase lipase, leucine arylamidase, valine arylamidase, α-chymotrypsin, β-galactosidase, β-glucosidase and naphthol-AS-BI-phosphohydrolase; does not express amylase, gelatinase, indole, lipase, urease, arginine dihydrolase, gelatinase, amylase, α-galactosidase, β-glucuronidase, α-glucosidase, α-mannosidase, *N*-acetyl-β-glucosaminidase and α-fucosidase. The strain hydrolyses aesculin, but not Tweens 20, 40, 60 or 80. Strain FW153^T^ uses acetate, bactopeptone, butyrate, casamino acids, glucose, glutamate, pyruvate, sucrose and yeast extract as a carbon source, but not adipic acid, arabinose, capric acid, citrate, ethanol, formate, fructose, potassium gluconate, glycerol, glycine, lactose, lactate, maltose, malate, mannose, mannitol, methanol, *N*-acetyl-d-glucosamine, phenylacetic acid, sorbitol and succinate. Major fatty acids are C_18 : 1_ ω7c, C_16 : 1_ and C_16 : 0_. The genome is 4.29 Mbp and has a G+C content of 65.54 mol%.

The type strain is FW153^T^ (=NCIMB 15610^T^, =DSM 120042^T^). It was isolated from the water of a marsh located in Winnipeg, Manitoba, Canada. The GenBank accession numbers for the 16S rRNA gene sequence and whole-genome sequencing project of FW153^T^ are PX123967 and JBLTWO000000000, respectively.

## Description of *Sphingomonas eleionomae* sp. nov.

*Sphingomonas eleionomae* (e.lei.o.no'mae. Gr. fem. n. *Eleionome*, nymph of marshes, ponds and wetlands; N.L. gen. n. *eleionomae*, of Eleionome; referring to the marsh isolation environment).

Cells are Gram-negative, motile rods that are ~2.8–4.4 µm in length and 0.6–0.7 µm in width. The strain forms yellow, circular (1–2 mm), flat, translucent and shiny colonies with a soft texture when grown on RO plates for a week. It is obligately aerobic and heterotrophic. Growth occurs in the following conditions (optimum): between 20 and 41 °C (32 °C), pH of 6–10 (6) and NaCl (% w/v) up to 4.5% (0%). Bchl *a*, carotenoids, photosynthetic reaction centre and light-harvesting I complex are produced, indicative of aerobic anoxygenic photosynthesis. RuBisCo was not detected in the genome. Strain FW199^T^ produces oxidase, catalase, amylase, trypsin, acid phosphatase, alkaline phosphatase, esterase lipase, leucine arylamidase, valine arylamidase, α-chymotrypsin, β-galactosidase, α-glucosidase, β-glucosidase, *N*-acetyl-β-glucosaminidase and naphthol-AS-BI-phosphohydrolase; does not express nitrate reductase, gelatinase, indole, lipase, urease, arginine dihydrolase, cystine arylamidase, α-galactosidase, β-glucuronidase, α-mannosidase and α-fucosidase. The strain hydrolyses aesculin and Tweens 20, 60 or 80, but not Tween 40. FW199^T^ can use arabinose, bactopeptone, casamino acids, fructose, glucose, glutamate, lactose, maltose, mannose, *N*-acetyl-d-glucosamine, sucrose and yeast extract as a carbon source, but not acetate, adipic acid, butyrate, capric acid, citrate, ethanol, formate, potassium gluconate, glycerol, glycine, lactate, malate, mannitol, methanol, phenylacetic acid, pyruvate, sorbitol and succinate. Major fatty acids are C_18 : 1_ ω7c, C_16 : 1_ and C_16 : 0_. The genome is 3.09 Mbp and has a G+C content of 65.43 mol%.

The type strain is FW199^T^ (=NCIMB 15611^T^, =DSM 120043^T^). The habitat is marsh water located in Winnipeg, Manitoba, Canada. The GenBank accession numbers for the 16S rRNA gene sequence and whole-genome sequencing project of FW199^T^ are PP726896 and JBLTWP000000000, respectively.

## Supplementary material

10.1099/ijsem.0.007018 Uncited Supplementary Material 1.

## Abbreviations

AAP: aerobic anoxygenic phototrophs
ANI: average nucleotide identity
Bchl *a*: bacteriochlorophyll *a*
dDDH: digital DNA–DNA hybridization
PM: potato media
RO: rich organic
TE: trace elements
VS: vitamin solution
