# Supplementary material for: Rhizorhabdus antheiae sp. nov. and Sphingomonas eleionomae sp. nov., new aerobic anoxygenic phototrophs isolated from a Manitoban marsh
Source: Int J Syst Evol Microbiol. 2026 Jan 9;76(1):007018. doi: 10.1099/ijsem.0.007018 (PMC12785184; doi:10.1099/ijsem.0.007018)
Supplement: Uncited Supplementary Material 1. [file ijsem-76-07018-s001.pdf]

Supplementary Data

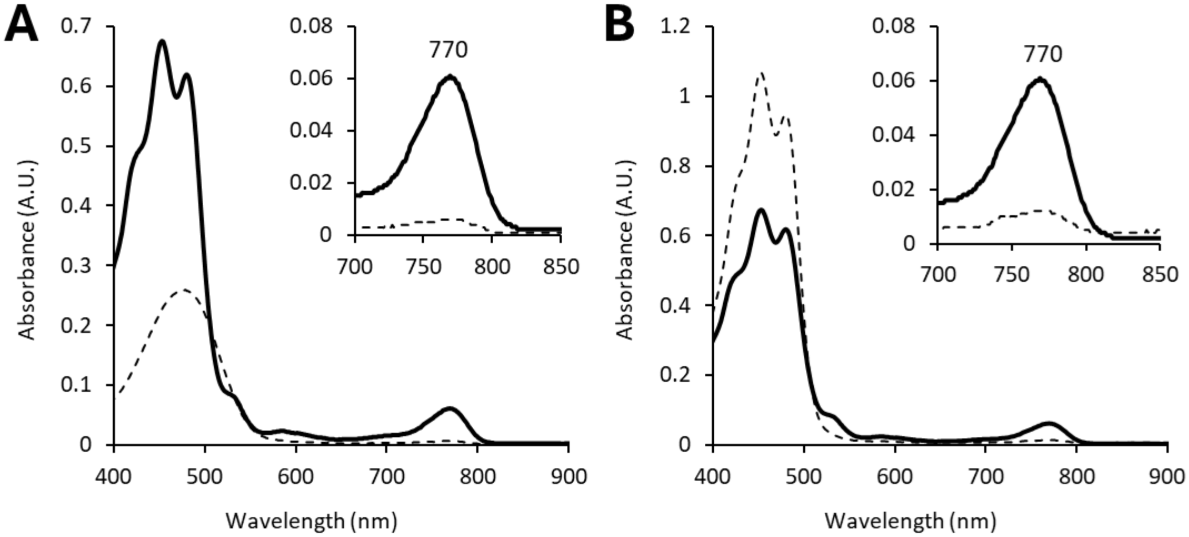

Figure S1. Absorbance spectra of pigment extracts of FW153^T (A) and FW199^T (B) grown in the dark (bold line) and in the light (dashed line). Wavelengths where significant peaks are observed are indicated.

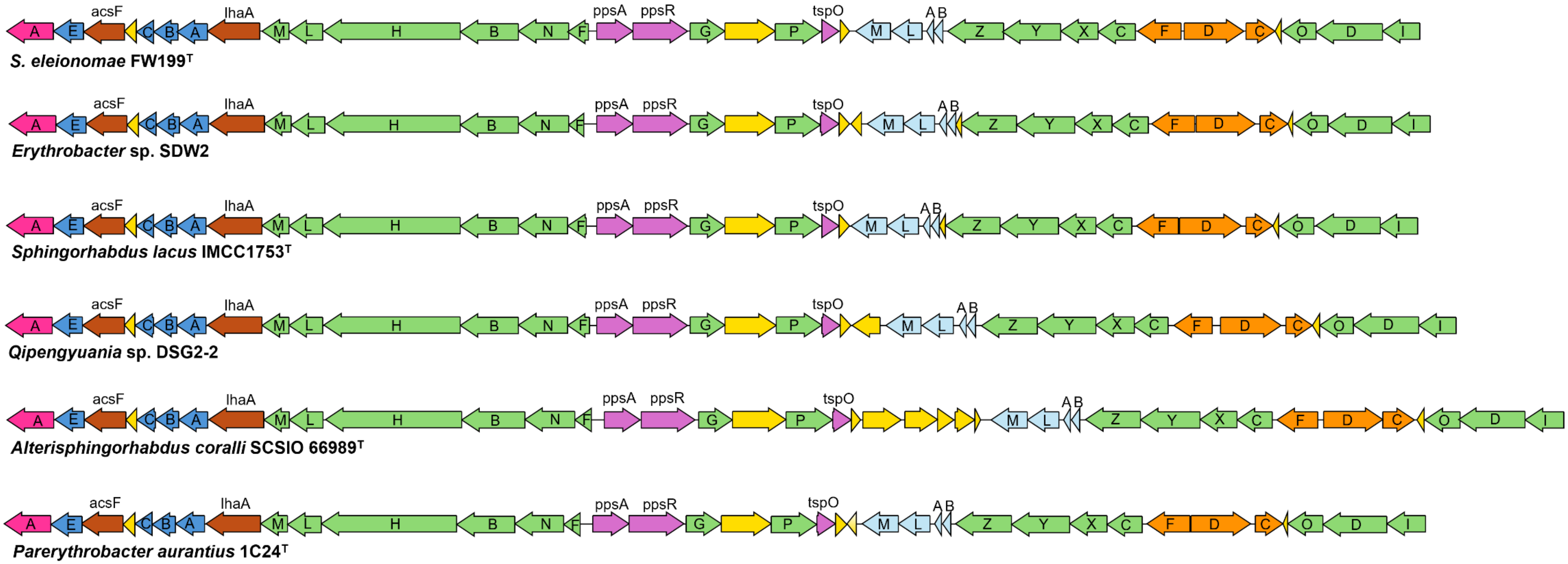

Fig. S2. The photosynthetic gene cluster of FW199^T compared to AAP with a similar organization. Genes are colored as follows: Green (*bch*), orange (*crt*), light blue (*puf*), blue (*puh*), pink (*hem*) purple (regulatory genes), brown (other photosynthesis genes), yellow (hypothetical proteins, uncertain or unrelated genes). GenBank genome accession numbers of strains (starting at top row): GCA_049889035.1, GCA_021431965.1, GCA_009768975.1, GCA_042492175.1, GCA_032852305.1, GCA_045291475.1.

Table S1. Fatty acid composition of FW153^T and FW199^T.

**Species**	***R. antheiae***	***S. eleionomae***
**Strains**	**FW153^T**	**FW199^T**
**Fatty acid composition (%)**		
$C_{14:0}$	0.9	0.6
$C_{15:0}$	0.6	0.7
$C_{16:0}$	14.4	6.7
$C_{16:1}$	25.8	16.9
$C_{16:1}$ t	0.2	0.2
$C_{17:0}$	0.2	0.1
$C_{17:1}$	0.3	0.7
$C_{18:0}$	0.6	0.1
$C_{18:1}$	6.3	0.8
$C_{18:1}$ ω7c	50.8	73.2
